# Enhancement of task-switching performance with transcranial direct current stimulation over the right lateral prefrontal cortex

**DOI:** 10.1007/s00221-021-06212-7

**Published:** 2021-09-12

**Authors:** Kristin Prehn, Anja Skoglund, Tilo Strobach

**Affiliations:** Department of Psychology, MSH Medical School Hamburg – University of Applied Sciences and Medical University, Am Kaiserkai 1, 20457 Hamburg, Germany

**Keywords:** Task switching, Executive control, Non-invasive brain stimulation, Transcranial direct current stimulation (tDCS), Right lateral prefrontal cortex (lPFC)

## Abstract

Switching between two or more tasks is a key component in our modern world. Task switching, however, requires time-consuming executive control processes and thus produces performance costs when compared to task repetitions. While executive control during task switching has been associated with activation in the lateral prefrontal cortex (lPFC), only few studies so far have investigated the causal relation between lPFC activation and task-switching performance by modulating lPFC activation. In these studies, the results of lPFC modulation were not conclusive or limited to the left lPFC. In the present study, we aimed to investigate the effect of non-invasive transcranial direct current stimulation [tDCS; anodal tDCS (1 mA, 20 min) vs. cathodal tDCS (1 mA, 20 min) vs. sham tDCS (1 mA, 30 s)] over the right inferior frontal junction on task-switching performance in a well-established task-switching paradigm. In response times, we found a significant effect of tDCS Condition (atDCS, ctDCS vs. sham) on task-switching costs, indicating the modulation of task-switching performance by tDCS. In addition, we found a task-unspecific tDCS Condition effect in the first experimental session, in which participants were least familiar with the task, indicating a general enhancement of task performance in both task repetitions and task-switching trials. Taken together, our study provides evidence that the right lPFC is involved in task switching as well as in general task processing. Further studies are needed to investigate whether these findings can be translated into clinically relevant improvement in older subjects or populations with executive function impairment.

## Introduction

Task switching describes the capacity to shift between the processing of two or more cognitive tasks. While allowing to adapt to different situations rapidly and efficiently, task switching usually leads to slowed and erroneous information processing. These *task-switching costs* are assumed to be caused by higher cognitive demands due to executive control processes which are, for instance, necessary to inhibit the previous and activate the subsequent task set (Monsell 2003; Yeung and Monsell 2003; Koch and Allport 2006; Kiesel et al. 2010).

To measure task-switching costs in the laboratory, participants are typically requested to perform two different tasks sequentially. For example, participants are presented with digits and indicate by right and left button press whether a digit is even or odd in task condition A. In task condition B, subjects indicate whether the digit is higher or lower than a reference value. The required task is given by a prespecified task sequence (e.g., AABBAABB etc.). The task-switching costs that arise from that sequence (i.e., the “alternating-run paradigm”) are defined by the difference in response times (RTs) and/or error rates in task-switching trials (A after B and B after A) and task repetitions (A after A and B after B; Monsell 2003).

Recent functional magnetic resonance imaging (fMRI) studies have investigated the neural correlates associated with higher cognitive demands during task switching (e.g., Dove et al. 2000; Brass and von Cramon 2002, 2004; Luks et al. 2002; Braver et al. 2003). These studies consistently reported increased activation in a neural network centered on the inferior frontal junction (IFJ; for a review and a metaanalysis, see Brass et al. 2005; Derrfuss et al. 2005). Providing complementary evidence, neuropsychological studies in patients with focal brain lesions seem important to narrow down hypotheses on the localization of specific subcomponents involved in task switching (such as inhibition, activation, and maintenance of task sets; Shallice et al. 2008). Aron et al. (2004), for instance, compared patients with left/right frontal lesions and reported that both groups show greater task-switching costs than controls, which may have arisen, however, for different reasons: patients with right frontal lesions seem to suffer from impaired inhibition of inappropriate responses or task sets (Aron and Poldrack 2006), whereas patients with left frontal lesions demonstrate a more general deficit in task set configuration (i.e., they have difficulties to implement and become acquainted with the rules of a given task; see also Shallice et al. 2007).

In addition to lesion studies, non-invasive brain stimulation techniques, such as transcranial direct current stimulation (tDCS), offer the opportunity to investigate brain-behavior relationships in healthy subjects by a transient modulation of cortical excitability. tDCS involves the application of two electrodes (anode and cathode) on the scalp of the participant. A continuous weak current (0.5–2.0 mA) is applied and flows from anode to cathode modulating the neural activity underneath the electrodes. Underneath the anode, “anodal” tDCS (atDCS) with currents of 1 mA leads to a *depolarization* of neuronal populations causing excitatory effects, whereas underneath the cathode “cathodal” tDCS (ctDCS) causes *hyperpolarization* and thus inhibition of cortical neurons (Nitsche and Paulus 2000). It has to be noted that most of the studies investigating the mechanisms of tDCS were conducted by stimulating the primary motor cortex (M1). Therefore, it is not clear to what extent these findings are transferable to other brain areas (e.g., the prefrontal cortex) and cognitive functions that are supported by richer brain networks. In particular, the common assumption of a dual-polarity effect (i.e., an anodal-excitation and cathodal-inhibition) has not always been confirmed; while most studies found excitatory effects following atDCS, which can be measured by enhanced task performance, cathodal stimulation does not always cause inhibition (Jacobson et al. 2012). Finally, a/ctDCS can be compared with a “sham tDCS” condition, during which the current is ramped up and down already after a short period of time at the beginning of the experiment (e.g., 30 s). Sham tDCS does not lead to stimulation effects but the same minimal discomfort, which is hardly distinguishable from real stimulation by the participant and thus allows efficient blinding (Gandiga et al. 2006).

Only few studies so far have tried to modulate neural activity of the lateral prefrontal cortex (lPFC) with tDCS during task switching (Strobach and Antonenko 2017). Leite et al. (2013) used a bilateral (cross-hemispheric) frontal electrode montage and found that left atDCS (with cathode over the right homologue) compared to right atDCS (with cathode over the left homologue) and sham increased task-switching performance in one experimental paradigm (letter/digit naming task). Using another task (vowel-consonant/parity task), in contrast, they found that left atDCS decreased performance compared to the other two stimulation conditions. Pointing towards different roles for the left and right hemisphere in task switching, this study, however, did not provide conclusive evidence whether atDCS, ctDCS, or the combination of both can influence task-switching in a task-unspecific way. In a previous study of our group, Strobach and colleagues (2016) used unihemispheric tDCS over the left IFJ during a letter-digit task. In this task, letter-digit pairs were presented. The letter could be a consonant (G, K, M, R) or vowel (A, E, I, U) and the digit could be even (2, 4, 6, 8) or odd (3, 5, 7, 9). Subjects were instructed to either respond to the letters (i.e., to indicate by button press whether the letter was a consonant or vowel; letter task) or digits (i.e., to indicate whether the digit was even or odd; digit task) in a prespecified sequence (e.g., AABBAABB etc.) in an alternating runs paradigm, which resulted in task repetitions alternating with task switches (Rogers and Monsell 1995). This study showed an unspecific atDCS effect at a low level of task automization (i.e., only at the first experimental session), which might be related to increased task set maintenance. Beyond this effect, however, the study was not able to show any effect of tDCS applied over the left IFG on task-switching performance.

Following these results of the previous tDCS studies and the possible dissociation between left and right frontal cortex function during task switching suggested by Aron et al. (2004), tDCS over the left IFG may only target some general processes of task set configuration and maintenance (i.e., processes which enable the participant to implement and become acquainted with the task). TDCS over the right IFG, in contrast, could more specifically modulate executive control necessary for the inhibition of the previous task set (see also Aron and Poldrack 2006) and thus might result in greater effects on task-switching performance.

In the present study, we investigated the causal relation between *right* lPFC activation and task-switching performance in the letter-digit task by modulating the neural activity of the right IFG with atDCS, ctDCS, and sham tDCS. We expected reduced task-switching costs (calculated by the difference in RTs and error rates in task-switching and task repetition trials) during atDCS, while task-switching costs should be increased during ctDCS.

## Materials and methods

### Study overview

The study employed a sham-controlled, single-blind within-subjects design and was conducted at the Institute of Research and Education GmbH (IRE) at MSH Medical School Hamburg. Thirty-six participants completed a well-established experimental task-switching paradigm (i.e., the letter-digit task) during tDCS over the right IFG. As indicators of task performance, we recorded RTs (primary outcome) and error rates. Each subject received all three tDCS conditions: atDCS, ctDCS, and sham tDCS in three experimental sessions. The order of tDCS conditions was balanced in a Latin square logic. To avoid cumulative tDCS effects, experimental sessions were separated by a wash-out phase of approximately one week (interval between Session 1 & 2: *M* = 6.7 days, *SD* = 1.4, range: 5–11 days; interval between Session 2 & 3: *M* = 7.2 days, *SD* = 1.4, range: 4–10 days).

### Participants

Thirty-six participants (mean age: 23.3 years [*SD* = 3.4, range: 19–32]; 30 women) took part in the study. All participants were bachelor or master students in Psychology at MSH Medical School Hamburg, right-handed (according to the Edinburgh handedness inventory (Oldfield 1971; mean handedness score: 91.4, SD = 18.3), had normal or corrected-to-normal vision, and were fluent in German and naïve to the purpose of the experiment. From the 36 participants, four subjects (all women) did not complete the study for personal reasons (i.e., time constraints). Unfortunately, data of two additional women could not be analyzed due to technical problems during recording, leaving the data of 30 subjects for the statistical analysis.

### Letter-digit task

**Figure1:**
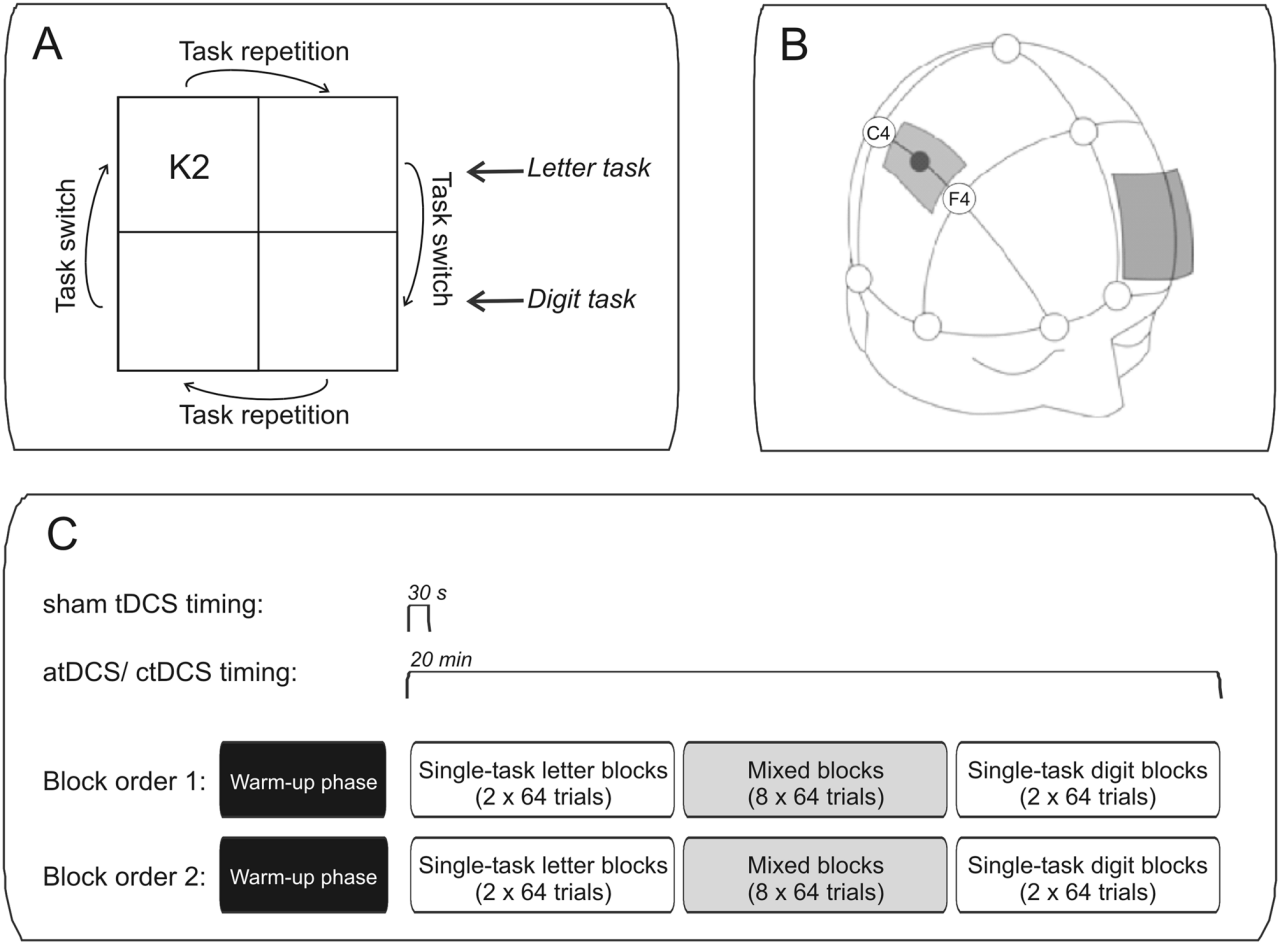
Study methods. **A** Illustration of a stimulus of the letter-digit task. Letter-digit pairs were sequentially presented and rotated clock-wise from box to box (arrows illustrate this rotation). Participants were instructed to respond to letters (letter task: consonant vs. vowel) or digits (number task: even number vs. odd number) when pairs were presented in the upper two or lower two boxes, respectively. This instruction resulted in situations with task repetitions and task switches. **B** Illustration of the location of the stimulation electrode (right inferior frontal gyrus, IFG; between F4 and C4) as well as the reference electrode. **C** Illustration of the experimental course and timing of the three transcranial direct current stimulation (tDCS) conditions: anodal, cathodal and sham tDCS. Stimulation was initiated in all conditions after a warm-up phase and at the beginning of the first single-task letter/digit block

To investigate task-switching performance, we used a letter-digit task already used in our previous study (Rogers and Monsell 1995; Strobach et al. 2016). In this task, letter-digit pairs were presented in one of four boxes as depicted in Fig. 1A. The letter could either be a consonant (G, K, M, R) or a vowel (A, E, I, U) and the digit could either be even (2, 4, 6, 8) or odd (3, 5, 7, 9). Participants were instructed to respond to the letter when the letter-digit pair was presented in one of the upper left and right boxes (letter task) and instructed to respond to the digit when the pair was presented in one of the lower boxes, repectively (digit task). In particular, participants pressed a left key with the left index finger when a consonant or an even digit was presented and a right key with the right index finger when a vowel or an odd digit was presented. Presentation of the first stimulus pair in each block started in the upper left box and the trial-to-trial presentation moved clockwise to the subsequent box. Since stimulus presentation rotated clockwise from box to box, task instruction resulted in situations with task repetitions (i.e., repeat trials) alternating with task switches (i.e., task-switching trials; see Fig. 1A).

### Transcranial direct current stimulation

The stimulation protocol was similar to that of previous studies from our group (e.g., Strobach et al. 2016, 2018; Prehn et al. 2017). A constant direct current (1 mA) was administered by a battery-driven stimulator (DC-Stimulator, NeuroConn GmbH, Ilmenau, Germany) and two electrodes, which were inserted into synthetic sponges soaked with saline solution and attached to the scalp using rubber bands. The stimulating electrode (to which the terms “atDCS” and “ctDCS” refer, 5 × 7 cm) was centered over the right IFJ [cortical coordinates: exactly half-way between F4 and C4 (position delineated according to the international 10–20 EEG system; DaSilva et al. 2011), see Fig. 1B]. The reference electrode (10 × 10 cm) was placed over the left supraorbital region. The size of the reference was chosen to be larger than the size of the anode/cathode to render its stimulation functionally inefficient (Nitsche et al. 2007). In atDCS and ctDCS conditions, a constant direct current of 1 mA was applied for 20 min. The current was increased over 10 s at the beginning of the stimulation (fade in) in a ramp-like fashion eliciting a tingling sensation on the scalp and decreased over 5 s at the end (fade out). During sham stimulation, the procedure was essentially the same with the only difference being that the current was ramped down automatically already after 30 s.

### Experimental procedures

The experiment took place in a quiet room at the IRE laboratories. Before the first experimental session, study exclusion criteria (e.g., history of chronic or acute neurologic, psychiatric, or serious medical disease) were carefully checked. Participants were then instructed and tDCS electrodes were placed on the participants’ head. Participants were seated comfortably in front of a computer screen (with a distance of approximately 0.6 m) on which the letter-digit task was presented using a customized experimental control software (Presentation, Neurobehavioral Systems Inc., Albany, CA, USA) running on a Microsoft Windows operating system.

In each trial, the letter-digit pair appeared and remained on the screen until the participant pressed a response button or 5 s had elapsed. When the participant made a correct response, a blank interval of 150 ms followed before a new trial started. When the participant made a mistake, a beep sounded for 30 ms and the following inter-trial interval was extended to 1.5 s. Participants were instructed to respond as quickly and correctly as possible. Letters and digits as well as the order of both in the stimulus pair were randomly selected.

Trials were presented in two types of blocks: single-task and mixed-task blocks. In single-task blocks, participants either performed the letter or the digit task exclusively (i.e., single-task trials). In mixed-task blocks, however, participants were instructed to repeat or switch between letter and digit tasks depending on the position of the letter-digit stimulus pair on the screen.

The experiment began with a warm-up phase consisting of a short letter and a short digit single task block (64 trials in total; see Fig. 1C). Then, the experimental phase with tDCS started and one half of the participants conducted the letter task in two long single-task blocks (128 trial in total), followed by 8 long mixed blocks (512 trials in total) and two final long single-task blocks of the digit task (128 trials in total). The other half of participants was presented with a reversed order of the single-task blocks (i.e., two long digit-task block at the beginning and two final long letter-task blocks).

To assess whether tDCS conditions caused changes in mood and affect, participants completed two self-report rating scales before and after each session: the Visual Analog Mood Scales (VAMS; Folstein and Luria 1973) and the Positive and Negative Affect Scales (PANAS; Watson et al. 1988). The VAMS comprises eight items that assess positive (2 items: happy and energetic) and negative (6 items: afraid, confused, sad, angry, tired, and tense) mood using a visual analog scale (range: 0–1). The PANAS assesses positive and negative affect (10 items each; positive: active, interested, excited, strong, inspired, proud, enthusiastic, alert, determined, attentive; negative: distressed, upset, guilty, scared, hostile, irritable, ashamed, nervous, jittery, afraid) on a scale ranging from 1 to 5. Higher values on VAMS and PANAS scales indicate more positive or negative mood and affect ratings.

At the end of the last experimental session, participants gave self-reports on side effects related to each stimulation session on a four-point scale (1 = no side effects to 4 = strong side effects) in the following criteria: headache, neck pain, pain on scalp, tickling, itching, burning, reddened skin, fatigue, poor concentration, and acute mood swings. They also indicated how much they believed that side effects were caused by tDCS on a five-point rating scale (1 = not caused by tDCS to 5 = clearly caused by tDCS). Finally, participants were debriefed about the different tDCS conditions.

### Statistical analyses

In a first step, we analyzed potential effects of tDCS on side effects, as well as on positive/negative mood and affect. In detail, a multivariate analysis of variance (MANOVA) across the different types of possible side effects and tDCS conditions was used. Positive/negative affect and mood ratings assessed with VAMS and PANAS before and after each experimental session were compared using repeated measures analyses of variance (ANOVAs) with the within-subject factors tDCS Condition (atDCS, ctDCS, vs. sham tDCS) and Time (before vs. after stimulation).

Data of the task-switching paradigm (RTs = primary outcome, error rates) were collapsed across digit and letter tasks. From RT analyses, we excluded trials with erroneous responses and trials with RTs 2 *SD* above the mean. Because RTs differed significantly between task conditions (single task, task repetition, and task switches), the boundaries for outliers were calculated for each condition separately.

As a manipulation check analysis, we analyzed whether there was a significant difference between in RTs and error rates between task switching vs. repetition trials (averaged across the different stimulation conditions) using repeated-measures ANOVAs for RTs and error rates with the factor Task (repetition vs. task-switching trials).

We then computed task-switching costs by calculating the difference in RTs and error rates between task-switching and repetition trials (RT and error rate switching cost scores) and analyzed differences in these scores between the three stimulation conditions: atDCS, ctDCS, and sham tDCS using repeated measures ANOVAs with the factor tDCS Condition. In the RT data analysis, mean single task RTs for each subject obtained in the two single task blocks (presented at the beginning and the end of the experiment) were entered as a covariate to control for individual differences in task processing speed.

Since we investigated tDCS effects in a within-subjects approach, all participants received all three tDCS conditions and performed the task-switching paradigm in three consecutive experimental sessions. To account for practice effects occurring over the course of the three experimental sessions (which have also been reported in previous studies using this experimental paradigm, see Strobach et al. 2016), we estimated effects of Session Number (1, 2 vs. 3) and Session Half (first vs. second half) on RT switching cost scores, following a between-subjects approach (ANOVA with the factors tDCS Condition, Session Number, and Session Half). Finally, we analyzed tDCS stimulation effects on RT data in the first and third sessions separately.

For significant main effects and interactions, we report partial *η*^2^ (partial eta squared) and exploratory post hoc *t* tests were performed to further characterize significant effects. All tests were two tailed, and the significance threshold was set at *p* < 0.05. All statistical analyses were performed using SPSS 25.0 (SPSS; IBM, Armonk, NY, USA).

## Results

### Side effects of tDCS administration and effects on positive/negative mood and affect

All subjects tolerated tDCS administration well. No serious adverse events were reported. Self-reported side effects (i.e., headache, neck pain, pain on scalp, tickling, itching, burning, reddened skin, fatigue, poor concentration, and acute mood swings), as assessed for each session after the end of the last session, showed no effects [*F*(20, 158) = 0.44, *p* = 0.98; see Table 1].

**Table 1:** Side effects [mean (standard error of the mean); range: 1–4] for “anodal” transcranial direct current stimulation (tDCS), “cathodal” tDCS, and “sham” tDCS (atDCS, ctDCS, and sham) as reported by the participants after the last experimental session. Higher values indicate higher intensity of side effects

Self-reported side effects	atDCS	ctDCS	sham tDCS
Headache	1.70 (0.17)	1.47 (0.13)	1.33 (0.11)
Neck pain	1.30 (0.12)	1.2 (0.10)	1.23 (0.11)
Pain on scalp	1.30 (0.13)	1.30 (0.11)	1.17 (0.07)
Tickling	1.57 (0.14)	1.60 (0.13)	1.53 (0.14)
Itching	1.73 (0.19)	1.57 (0.15)	1.57 (0.16)
Burning	1.80 (0.16)	1.70 (0.15)	1.53 (0.13)
Reddened skin	1.10 (0.07)	1.07 (0.07)	1.03 (0.03)
Fatigue	1.70 (0.17)	1.60 (0.17)	1.73 (0.15)
Poor concentration	1.53 (0.15)	1.50 (0.13)	1.37 (0.13)
Acute mood swing	1.13 (0.10)	1.07 (0.07)	1.17 (0.12)

We did not explicitly asked participants to indicate the individual order of tDCS conditions after debriefing. However, participants indicated for each reported side effect how much they thought it was caused by tDCS. Since causality ratings did not differ between tDCS conditions in a repeated-measures ANOVA with the within-subject factor tDCS Condition [*F*(2, 58) = 0.98 *p* = 0.38], we conclude that tDCS-related blinding was successful.

The values of VAMS and PANAS assessing positive/negative mood and affect are given in Table 2 (higher values indicate more positive or more negative mood and affect ratings). There was no main effect or interaction with the factor tDCS Condition on VAMS’ positive/negative mood ratings [*F*s(2, 58) < 1.92, *p*s > 0.16/*F*s(2, 58) < 2.50, *p*s > 0.09]. There was also no main effect or interaction with the factor tDCS Condition on the negative affect scale of the PANAS [*F*s(2, 58) < 2.82, *p*s > 0.07]. On ratings of the positive scale, however, we found a statistically significant tDCS Condition x Time interaction [*F*(2, 58) = 3.44, *p* = 0.04, partial *η*^*2*^ = 0.11]. This interaction results from the particular more positive affect rating in the ctDCS condition compared to the other two stimulation conditions already before stimulation (see Table 2). Thus, potential effects of tDCS on performance in the task-switching paradigm cannot be explained by differences in self-reported side effects, mood and positive/negative affect.

**Table 2:** Positive and negative mood and affect ratings [mean (standard error of the mean)] as measured with the Visual Analog Mood Scales (VAMS, range: 0–1) and the Positive and Negative Affect Scales (PANAS, range: 1–5) before and after “anodal” transcranial direct current stimulation (tDCS), “cathodal” tDCS, and “sham” tDCS (atDCS, ctDCS, and sham). Higher values indicate more positive or negative mood and affect ratings

	Before stimulation			After stimulation		
	atDCS	ctDCS	sham	atDCS	ctDCS	sham
VAMS						
Positive score	1.45 (0.13)	1.67 (0.11)	1.47 (0.12)	1.30 (0.15)	1.35 (0.14)	1.40 (0.14)
Negative score	0.55 (0.08)	0.46 (0.05)	0.53 (0.06)	0.50 (0.06)	0.51 (0.07)	0.45 (0.05)
PANAS						
Positive score	2.81 (0.12)	3.01 (0.13)	2.72 (0.12)	2.66 (0.14)	2.66 (0.13)	2.69 (0.15)
Negative score	1.33 (0.08)	1.18 (0.03)	1.27 (0.07)	1.22 (0.04)	1.19 (0.07)	1.11 (0.03)

### tDCS effects on RTs and error rates

First, we analyzed in a manipulation check analysis whether there was a significant difference in RTs and error rates between task-switching vs. repetition trials (averaged across the different stimulation conditions). As predicted, this analysis revealed that participants were slower and made more errors in task-switching trials compared to task repetitions [RTs: *F*(1, 29) = 572.41, *p* < 0.001, partial *η*^*2*^ = 0.95, M_repeat_ = 762 ms, SD_repeat_ = 118 ms, M_switch_ = 1258 ms, SD_switch_ = 177 ms; error rates: *F*(1, 29) = 72.71, *p* < 0.001, partial *η*^*2*^ = 0.72, M_repeat_ = 0.03, SD_repeat_ = 0.04, M_switch_ = 0.06, SD_switch_ = 0.05].

**Fig. 2:**
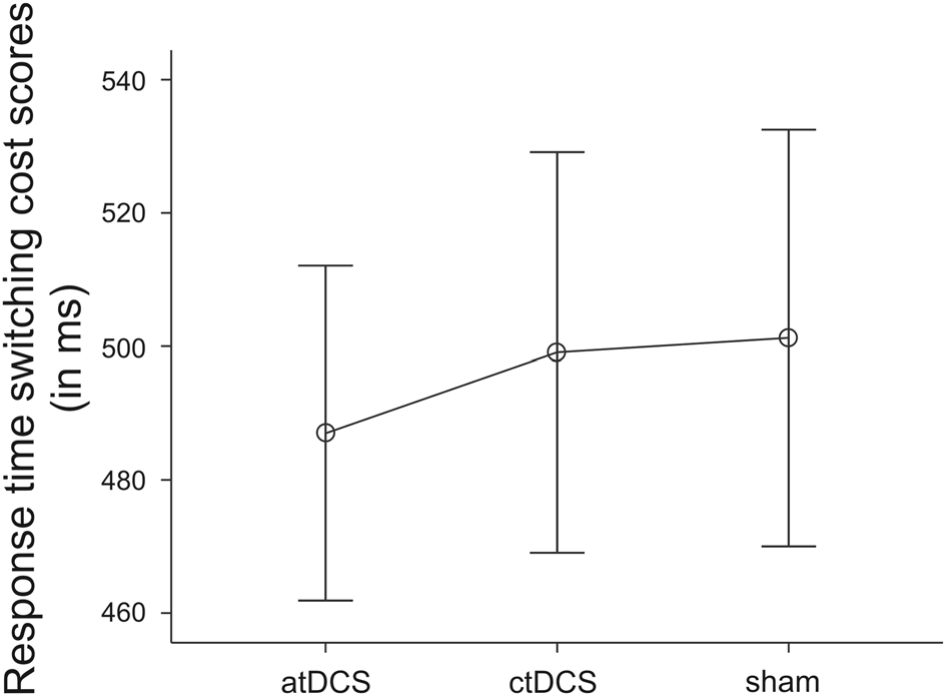
Response time switching cost scores (mean and standard error of the mean in ms) for “anodal” transcranial direct current stimulation (tDCS), “cathodal” tDCS, and “sham” tDCS (atDCS, ctDCS, and sham)

A repeated-measures ANOVA on RT switching cost scores then revealed a significant effect of tDCS Condition [*F*(2, 56) = 3.50, *p* = 0.037, partial *η*^2^ = 0.11; atDCS: *M* = 486.98, SD = 137.60, ctDCS: *M* = 499.09, SD = 164.52, sham tDCS: *M* = 501.26, SD = 171.13]. As displayed in Fig. 2, RT task-switching costs were lowest during atDCS. Exploratory post hoc *t* tests comparing the costs between the three stimulation conditions, however, found no significant differences [*t*s(29) < 0.38, *p*s > 0.71].

In error rates (error rate switching costs), we found no effect of tDCS Condition [*F*(2, 58) = 0.15, *p* = 0.865; atDCS: *M* = 0.034, SD = 0.028, ctDCS: *M* = 0.030, SD = 0.036, sham tDCS: *M* = 0.031, SD = 0.026].

### Practice effects on RTs

Since large practice effects across and within experimental sessions in the task-switching paradigm have been reported (Strobach et al. 2016), we estimated effects of Session Number and Session Half on RT switching cost scores. This analysis showed a significant effect of Session Number [*F*(2, 162) = 28.39, *p* < 0.001, partial *η*^2^ = 0.26], but no effect of Session Half [*F*(1, 162) = 0.62, *p* = 0.433]. Moreover, we found a significant Session Number x tDCS Condition interaction [*F*(4, 162) = 2.80, *p* = 0.028, partial *η*^2^ = 0.065]. An interaction of Session Half with tDCS Condition was not found [*Fs*(2, 162) = 0.97, *p* = 0.38].

**Fig. 3:**
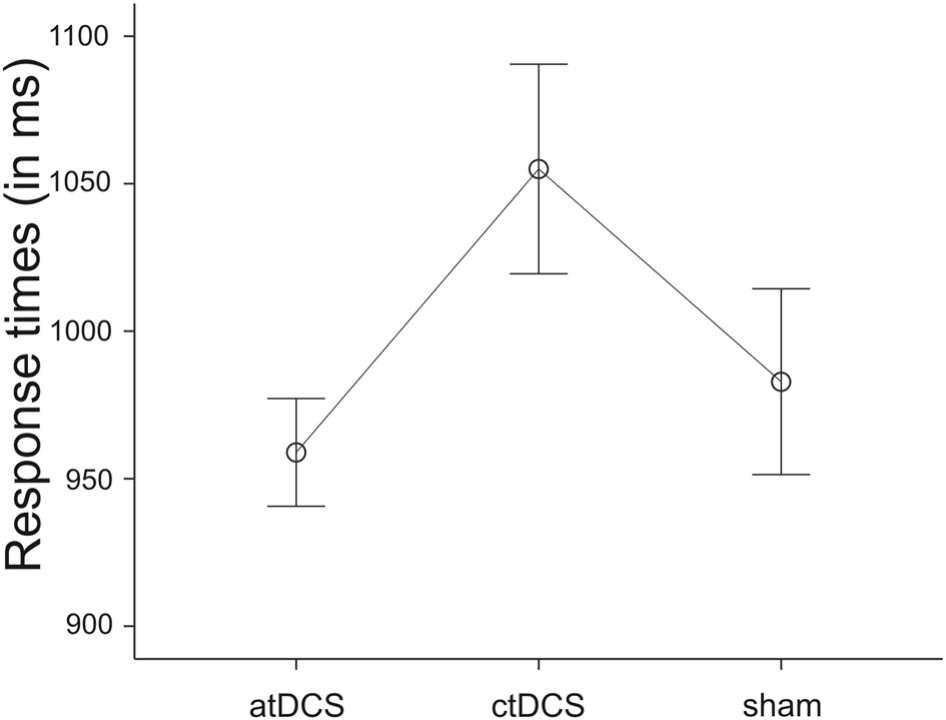
Response times (mean and standard error of the mean in ms) for task repetitions and switches taken together in session 1 for “anodal” transcranial direct current stimulation (tDCS), “cathodal” tDCS, and “sham” tDCS (atDCS, ctDCS, and sham)

Following up on the significant Session Number x tDCS Condition interaction in RT switching cost scores, we analyzed RT data for the first and third sessions separately. In the first session, in which subjects had no or least practice and longest RTs, there was no effect of tDCS Condition on RT switching cost scores [*F*(2) = 0.58, *p* = 0.56]. An ANOVA with the factors tDCS Condition and Task (repetition vs. task-switching trials), however, revealed a significant tDCS Condition effect [*F*(2, 54) = 3.65, *p* = 0.033, partial *η*^2^ = 0.12], a significant Task effect [*F*(2, 54) = 276.03, *p* < 0.001, partial *η*^2^ = 0.84], but no tDCS Condition x Task interation [*F*(2, 54) = 0.25, *p* = 0.78]. The significant tDCS Condition effect is displayed in Fig. 3.

Exploratory post hoc *t* tests revealed slower RTs during atDCS compared to ctDCS [*t*(17) = − 2.32, *p* = 0.03], whereas the other comparisons revealed no significant differences [atDCS vs. sham: *t*(18) = − 0.62, *p* = 0.54; ctDCS vs. sham: *t*(19) = 1.52, *p* = 0.14].

In the last session with shortest RTs, we also found no effect of tDCS Condition on RT switching cost scores [*F*(2) = 2.49, *p* = 0.103]. The ANOVA with the factors tDCS Condition and Task (repetition vs. task-switching trials) did also not reveal a significant tDCS Condition effect [*F*(2,54) = 0.52, *p* = 0.60], nor a tDCS Condition x Task interaction [*F*(2,54) = 0.56, *p* = 0.58].

## Discussion

In the present study, we aimed at investigating whether a tDCS-induced transient modulation of cortical excitability influences task-switching performance. Participants completed a well-established experimental task-switching paradigm (letter-digit task) during three sessions of tDCS (atDCS, ctDCS and sham tDCS) over the right IFJ. First, we found a significant tDCS effect on task-switching costs in RTs, indicating a modulation of task-switching performance by tDCS. Second, there was no evidence that modulation resulted from self-reported side effects or tDCS-related changes in mood and positive/negative affect. Third, by analyzing RT data only in the first experimental session, we found a task-unspecific tDCS effect (for both task repetitions and task-switching trials) with faster responses during atDCS, while responses were slowed down in the ctDCS conditions.

### tDCS-induced enhancement of task-switching performance

We found a significant effect of tDCS Condition on task-switching costs in RTs, indicating a modulation of task-switching performance by tDCS. In particular, task-switching costs were shortest during atDCS. Since post hoc *t* tests (which differed from the ANOVA by not taking individual differences such as the baseline covariate into account) failed to reveal significant differences between the three stimulation conditions, we very cautiously conclude that the upregulation of neural excitability in the right IFJ enhances task-switching performance. The result is in line with fMRI studies demonstrating the involvement of the IFJ/lPFC in cognitive control and task switching (Brass et al. 2005; Derrfuss et al. 2005).

In contrast to our hypotheses, we did not find an effect of tDCS on task-switching costs in error rates. Error rates were generally low. That is, on average we found below 7% of errors in task-switching trials compared to 4% in task repetitions. The result, however, is in line with other studies (e.g., Strobach et al. 2016) showing that RT constitutes a more sensitive marker when investigating tDCS effects on task (switching) performance (for a review, see Strobach and Antonenko 2017).

The specific role of the right IFJ/lPFC in task switching, however, is still not clear and has to be investigated in further studies. Dove et al. (2000), for instance, investigated the neural correlates of task switching with fMRI and found that a number of brain regions, including the bilateral lPFC and premotor cortex, the bilateral anterior insula, the left intraparietal sulcus, the supplementary and pre-supplementary motor area as well as the bilateral cuneus/precuneus, was activated by both a task repetition and switching condition and showed additional activation during task switching. These results showed that the lPFC is neither the only brain region involved, nor a region specifically involved in task switching. Shallice and colleagues (2008) integrated the results of fMRI studies and studies in neurospychological patients into the conceptual framework of the Supervisory Attentional System (Shallice and Burgess 1996) and suggested a crucial role of the right lPFC in monitoring processes during task switching, such as the inhibition of an inappropriate task set, whereas the left lPFC seems to be more involved in task setting (i.e., activation and automatization of an appropriate task set). The idea that left and right lPFC have different functions in task switching is further supported by Strobach et al. (2016), who modulated neural activity of the left lPFC by tDCS and only found an unspecific atDCS effect related to increased task set maintenance. Wang and colleagues (2020), moreover, compared the effects of left and right anodal tDCS on predictable and unpredictable task-switching situations and also found evidence for a stronger atDCS effect over the right DLPFC in unpredictable but not predictable task switching, which again has been interpreted as evidence for the role of the right lPFC in task-set inhibition (see also Wang et al. 2021).

It has to be noted that current concepts of dynamic hemispheric asymmetries go beyond and challenge the conservative notion of lateralization in task switching (Tayeb and Lavidor 2016). Using resting-state electroencephalography, Ambrosini et al. (2020), for instance, computed a lateralization index (i.e., a right-left difference score for the log-transformed power ratio between beta and alpha frequencies) for 75 homologous cortical regions and found that right lateralization in prefrontal brain regions was positively correlated with monitoring ability assessed with the performance in three different cognitive tasks. This result is in line with other studies (Smith et al. 2009; Mennes et al. 2011) showing that intrinsic brain dynamics already observable at rest, such as right lateralized resting state activity in nodes belonging to cognitive control and attention networks, may explain interindividual differences in executive functions.

There was no evidence in our study that the tDCS effect resulted from self-reported side effects or tDCS-related changes in mood and positive/negative affect. Since tDCS effects on task-switching costs, however, were rather small compared to the larger practice effects across the three experimental sessions, we analyzed RT data additionally in a between-subject approach for the first and third sessions seperately. In the first session, in which subjects had least practice (i.e., at the lowest level of task automization), we found a task-unspecific tDCS effect, indicating a general enhancement of task performance in both task repetitions and task-switching trials. The tDCS effect with faster processing during atDCS and slower processing in the ctDCS condition is well in line with the literature assuming that tDCS causes excitatory effects underneath the anode, while it causes hyperpolarization and inhibition of cortical neurons underneath the cathode (Nitsche and Paulus 2000), but does not support the hypotheses of the current study (which was the specific involvement of the right lPFC in task switching).

### Limitations of the present study

Some limitations should be considered when interpreting our findings. First, we cannot answer the question whether the results are specifically related to right IFJ function or whether stimulation of other brain regions would have resulted in similar effects. In addition to the limited spatial accuracy of tDCS, it should be noted that tDCS does not only affect the brain regions directly under the electrodes but may also modulate functional connectivity between remote but functionally related brain areas (Polania et al. 2011) or influence the within network connectivity (Meinzer et al. 2012). Using additional stimulation regions (e.g., the parietal cortex), however, would have improved the specificity of the results and should be considered in future studies.

Second, the sample size in our study was rather small; although comparable to sample sizes from previous studies (Leite et al. 2011, 2013; Strobach et al. 2016). While including 36 subjects in the study, we were only able to analyze the data of 30 subjects due to technical problems during data recording and four drop-outs. Therefore, we cannot fully exclude the possibility that a lack of statistically significant differences in task-switching costs between the three tDCS conditions (post hoc *t* tests) is due to the small number of participants. Thus, further studies are needed to replicate our results in larger samples.

Third, the analysis of RT data revealed that subjects improved over the course of the three experimental sessions. To rule out an influence of condition order, subjects were randomly assigned to different tDCS condition sequences. We also tested for a statistical interaction between the factors tDCS Condition, Session Number, and Session Half, which was not significant. However, we cannot exclude that the relatively small tDCS effects were overlaid by bigger practice effects. In future studies, a more comprehensive training/familiarization with the task before starting the intervention may dilute practice effects and provide a more reliable measure of change.

### Conclusion and outlook

The present study demonstrated that tDCS over the right lPFC modulates task-switching performance. However, the effect was only evident in RT task-switching costs (not in error rates) and could only revealed in the main analysis (an ANOVA when including a baseline covariate and not in the post hoc *t* tests comparing RT task-switching costs between atDCS, ctDCS, and sham stimulation). In addition, we found a task-unspecific tDCS Condition effect in the first experimental session, in which participants were least familiar with the task, indicating a general enhancement of task performance with atDCS in both task repetitions and task-switching trials. Therefore, we conclude that the right lPFC is involved in task switching as well as in general task processing. Although the results of the present study need to be replicated and evaluated in larger trials, we further believe that tDCS might offer exciting opportunities for enhancing executive control in older people and clinical populations which should be investigated in further studies.

## Declarations

### Availability of data and material

The datasets generated and/or analyzed during the current study are available from the corresponding author on reasonable request.

### Code availability

The code for running the experimental control software is available from the corresponding author on reasonable request.
